# FijiWings: An Open Source Toolkit for Semiautomated Morphometric Analysis of Insect Wings

**DOI:** 10.1534/g3.113.006676

**Published:** 2013-08-01

**Authors:** Alexander C. Dobens, Leonard L. Dobens

**Affiliations:** Division of Molecular Biology and Biochemistry, School of Biological Sciences, University of Missouri-Kansas City, Kansas City, Missouri 64110

**Keywords:** Drosophila, wing, growth, proliferation, ImageJ, Fiji, trichome, engrailed, posterior compartment

## Abstract

Development requires coordination between cell proliferation and cell growth to pattern the proper size of tissues, organs, and whole organisms. The Drosophila wing has landmark features, such as the location of veins patterned by cell groups and trichome structures produced by individual cells, that are useful to examine the genetic contributions to both tissue and cell size. Wing size and trichome density have been measured manually, which is tedious and error prone, and although image processing and pattern-recognition software can quantify features in micrographs, this approach has not been applied to insect wings. Here we present FijiWings, a set of macros designed to perform semiautomated morphophometric analysis of a wing photomicrograph. FijiWings uses plug-ins installed in the Fiji version of ImageJ to detect and count trichomes and measure wing area either to calculate trichome density of a defined region selected by the user or generate a heat map of overall trichome densities. For high-throughput screens we have developed a macro that directs a trainable segmentation plug-in to detect wing vein locations either to measure trichome density in specific intervein regions or produce a heat map of relative intervein areas. We use wing GAL4 drivers and UAS-regulated transgenes to confirm the ability of these tools to detect changes in overall tissue growth and individual cell size. FijiWings is freely available and will be of interest to a broad community of fly geneticists studying both the effect of gene function on wing patterning and the evolution of wing morphology.

Body size, a characteristic feature that distinguishes species, populations, and sexes, is influenced by both genetic and environmental factors (Edgar 2006; Mirth and Riddiford 2007). The size of tissues and organs is the outcome of an increase in both cell growth and proliferation during development, and these processes are regulated by interplay between (1) systemic signals that integrate nutritional and other influences to modulate the rate of tissue growth and (2) hormonal signals that control the length of the growth period (Caldwell *et al.* 2005; Colombani *et al.* 2005; Mirth *et al.* 2005).

Most cells double their mass during each division cycle, maintaining a roughly constant size as they proliferate. The classic studies of Hartwell in yeast (Hartwell 1971) showed that the cell cycle is linked to but regulated independently from cell growth (Johnston *et al.* 1977). In multicellular organisms, cell proliferation and growth is controlled further by (1) cell-cell contact (reviewed in Johnston and Gallant 2002); (2) short-range, tissue-specific−secreted growth factors, including members of the Wg/WNT, Dpp/BMP, Vn/EGF, Notch, Hippo, and Hedgehog pathways (Riddle *et al.* 1993; Struhl and Basler 1993; Zecca *et al.* 1995; Burke and Basler 1996; Chiang *et al.* 1996; Lecuit *et al.* 1996; Pagan *et al.* 1996; Chen and Struhl 1999); and (3) systemic insulin-like growth factor and steroid hormone signaling (Oldham and Hafen 2003). The crosstalk among these signals that regulates tissue size is an area of intense study (Strassburger *et al.* 2012).

During the past 15 years, researchers have used the fruit fly *Drosophila melanogaster* to understand how tissue size is achieved (reviewed in Mirth and Shingleton 2012). An excellent model tissue is the fly wing, which forms during the larval stages from an epithelial sheet that proliferates exponentially, roughly doubling in mass during a 10- to 12-hr period (Held 2002). The wing disk epithelium everts at metamorphosis to form the wing blade, which is marked by longitudinal and cross veins that provide structural strength and dotted with actin-rich trichome structures thought to confer aerodynamic properties. From wing to wing, both the shapes and positions of veins and the number and detailed orientation of trichomes are precisely patterned (De Celis 2003). Extensive work has identified many signaling pathways that pattern wing cell growth and proliferation (Crozatier *et al.* 2004; Shingleton 2005). Key among these are growth factor signals that regulate cell number to pattern the density of trichomes (Garcia-Bellido *et al.* 1994; Mata *et al.* 2000), and insulin signaling, which scales wing growth to the overall body size (Parker 2011). Mutations in the *chico* gene encoding the Insulin Receptor Substrate (IRS) result in a smaller body and correspondingly smaller wings (*e.g.*, Bohni *et al.* 1999).

The wing offers genetic tools, such as clonal analysis and region-specific GAL4 drivers, to perform screens for genes that control cell growth, cell-cycle progression, apoptosis, and terminal differentiation (*e.g.*, Gregory *et al.* 2007). In one example, wing cell clones mutant for dE2F have reduced numbers of larger cells whereas clones misexpressing dE2F have increased numbers of smaller cells (Neufeld *et al.* 1998). The ability to detect these complementary phenotypes in wing tissue led to the important conclusion that although cell growth and proliferation are linked, it is growth that typically drives wing cells through the cell cycle and not vice versa, just as in yeast (Conlon and Raff 1999).

Documenting precisely the effect of manipulating gene activity on wing differentiation is hampered by a paucity of tools to perform accurate morphometric measurements. Image analysis software has the potential to automate wing analysis; however, previous efforts have relied on expensive commercial software or specialized hardware (Houle *et al.* 2003). ImageJ, a software package provided by the U.S. National Institutes of Health (Schneider *et al.* 2012), can easily interface with data files produced by most automated microscopy systems to process and analyze objects within an image, and may be used on any computer capable of running Java platforms including Mac OS X, Linux x86 or Microsoft Windows. Here we present FijiWings, which can analyze the size and trichome distribution in photomicrographs of Drosophila wings and is implemented as a set of macros for the ActionBar plug-in added to Fiji (Fiji is a fully featured distribution of the popular ImageJ; Schindelin *et al.* 2012). We make the code of each macro available for further modifications and package them together in a version of Fiji that will allow ease of use.

## Materials and Methods

### Stocks

The stocks (1) {en2.4-GAL4}e16E (Brand and Perrimon 1994) and (2) P{TRiP.JF01859}attP2 (UAS-Pten RNAi; Perrimon *et al.* 2010) were obtained from the Bloomington Stock Center and (3) UAS-FLAGTrbl from our laboratory (Masoner *et al.* 2013). Crosses were reared at 30° to boost GAL4 activity.

### Wing preparation and microscopy

Proper wing preparation and photography is critical for success in wing morphometry. We removed wings from 3- to 8-d-old adult flies (here we examine females only) using forceps (Dumont #5 Forceps, Fine Science Tools; www.finescience.com) and transferred five to six wings to a drop of Euparal mounting medium (Bioquip.com) on a clean cover slip (Fisherbrand Superslip Cover Slips; Fisher Scientific). The slip was overlaid by a clean microscope slide (Fisherbrand Superfrost Plus; Fisher Scientific), the Euparal allowed to spread, then the sandwich was flipped right side up and pressure from a probe used to spread and separate the wings, which removed large bubbles as detected under the dissecting microscope. Slides were baked for 24 hr at 65° to slightly harden the Euparal and then weights (cored plug, red brass, 3/4-in; Grainger.com) were carefully laid on the cover slip to ensure flattening and further baked for another 24 hr. To visualize wing preparations, a Nikon TE-2000 with attached Colorview camera and Analysis image acquisition software microscope was used at either 200× or 40× with the condenser diaphragm fully closed to maximize contrast. After careful adjustment to focus on trichomes, photomicrographs (resolution 2080 × 1544) were collected.

### Macro description

**Figure 1:**
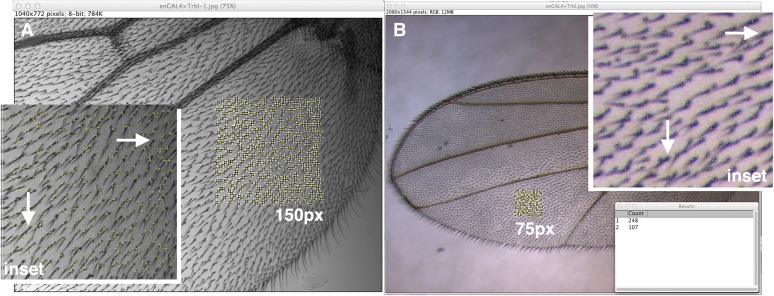
The 75px trichome density and 150px trichome density macros. (A) The “Find Maxima” function of Fiji/ImageJ is used to identify and count trichomes in 150px^2^ area selected by the user, here in the third posterior cell of a wing blade photographed at 200x. Inset shows close-up of this area and trichomes detected appear as yellow crosses. Top white arrow points to a trichome missed in the count, and bottom arrow points to two trichomes counted as one. (B) 75px^2^ area selection in the second posterior wing cell at 40x. Inset shows close-up of this area and trichomes detected appear as yellow crosses. Top white arrow points to a trichome missed in the count, and bottom arrow points to two trichomes counted as one.

**Figure 2:**
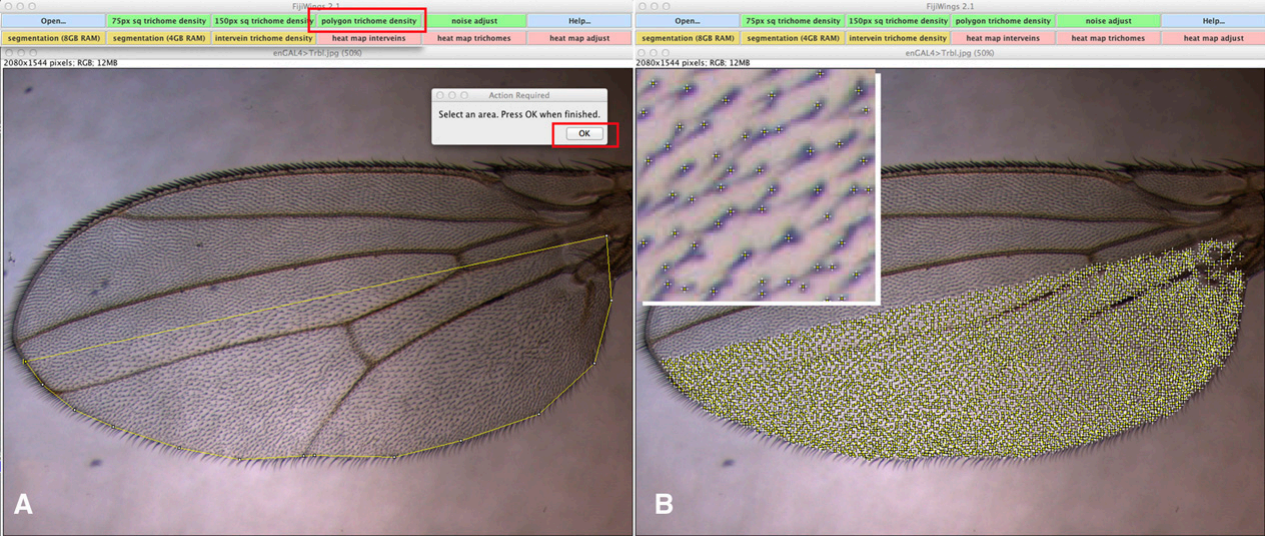
The polygon trichome density macro. (A) Work surface of Fijiwings with ActionBar at top. Boxed in red is the polygon trichome density button. Selecting this allows the user to outline a selected area in yellow, in this case the posterior compartment. Clicking the “OK” prompt (outlined in red, center) leads to the window in B. (B) After clicking “OK” in (A), the polygon is replaced by crosses, each marking a trichome detected in the selected area, in this case the entire posterior compartment. Inset shows close-up within this area and trichomes detected appear as yellow crosses.

**Figure 3:**
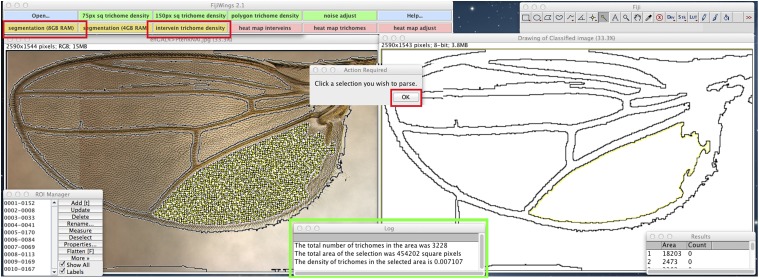
Identification of and determination of trichome density within intervein regions using the segmentation/intervein density macros. Work surface of FijiWings highlights **segmentation (8 GB)** button in ActionBar (outlined in red box, top). Clicking this button launches Weka segmentation analysis to identify wing vein and intervein regions and results in the presentation of the original micrograph (left panel) next to a “Drawing of Classified Image” (right panel). Clicking the **intervein trichome density** button (outlined in red, top), results in “Action Required” prompt. Manually selecting an intervein region in the “Drawing of Classified Image” by simply clicking within the outlined image leads to its highlighting in yellow (right panel). Subsequently clicking “OK” in the prompt (outlined in red, center) results in the identification and counting of trichomes in that selected intervein area (right panel), with data presented in “Log” window and information regarding area in the “Results” window.

**Figure 4:**
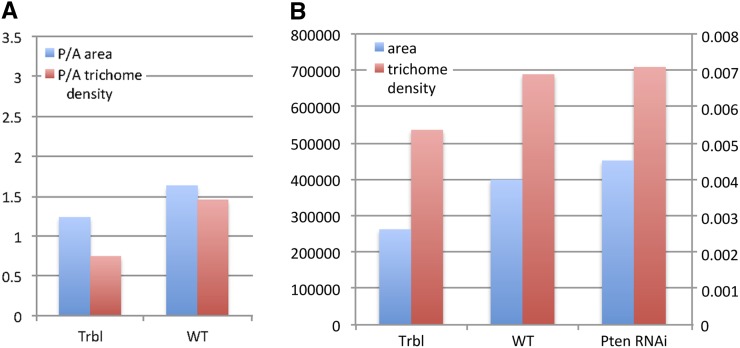
Trbl misexpression leads to reduced cell size and reduced tissue size while Pten RNAi misexpression leads to the complementary effect. (A) Graph comparing area (blue) and trichome density (red) ratios from measurements of posterior and anterior compartments from Trbl and WT wings, respectively. (B) Two-axis graph showing area (in pixels) and trichome density measurements (in trichomes per pixel) for third posterior cell from Trbl, WT, and Pten RNAi.

We used the Java code in Fiji (Schindelin *et al.* 2012) to write a set of macros linked to plug-ins that accomplish various and sundry measurements of wing properties and linked these macros to a set of labeled buttons (in **bold**, below) collected on an ActionBar toolbar (Figure 2; imagejdocu.tudor.lu/doku.php?id = plugin:utilities:action_bar:start), which appears at startup when FijiWings is launched as a series of buttons in two rows, from left to right:

(i) **Open**…
  This allows access to the directory of raw images.
(ii) **75px trichome density** By clicking on this button in the ActionBar, then selecting an area of the wing with the cursor and clicking on that spot, the tools uses the Process > Find Maxima function of Fiji/ImageJ to detect the dark base of each trichome in a 75px area. The resulting points (Figure 1B) are totaled in a “Results” window and the density (per pixel) calculation is presented in a “Log” window.
(iii) **150px trichome density**
  This button allows the counting of trichomes in a selected 150 pixel square.
(iv) **polygon trichome density**
  This function allows the user to select manually the entire compartment by a series of clicks (Figure 2A) and then calculates (1) the subtended area, (2) its total trichome count, and (3) the trichome density per pixel count and presents this information in a “Results” window (Figure 2B).
(v) **noise adjust**
  We empirically determined that when the “noise tolerance” is set at “10,” most trichomes are marked with a “point selection.” This selection allows the user to change this default setting.
(vi) **Help…**
  This links to the Wiki page for FijiWings.
(vii) **segmentation (8 GB)**
  This function uses a machine learning algorithm (Frank *et al.* 2004; reviewed in Shamir *et al.* 2010) to automatically identify and outline veins and wing edges in any micrograph and perform particle analysis of the output to outline each intervein region (and generate a region of interest [ROI] table). Clicking on the button initiates a macro that automatically (1) resizes the image to 512 × 380, (2) performs Weka segmentation analysis (Frank *et al.* 2004) to identify intervein regions, and then (3) resizes the resulting outline, placing it next to the original micrograph, which allows users to proceed to use the “**intervein trichome density**” or “**heatmap”** functions, discussed in the subsections to follow. Weka segmentation is RAM intensive and functions best on Macs with 4−8 GB of RAM.
(viii) **segmentation (4 GB)**
  This button opens another ActionBar that allows a step-wise performance of segmentation analysis. To summarize, STEP 1 resizes the image, STEP 2 performs Weka segmentation, STEP 3 produces an image of the Weka output, and STEP 4 resizes that image, allowing users to proceed to use the “**intervein trichome density**” or “**heatmap”** functions, discussed in the subsections to follow. We find this series of steps allows reliable segmentation on slower computers with 4 GB of RAM and has worked even on our older Macs with 2 GB of RAM by first increasing Java’s memory allotment (http://weka.wikispaces.com/OutOfMemoryException)
(ix) **intervein trichome density**
  Both **segmentation (8 GB)** (one step) and **segmentation (4 GB)** (four steps) functions result in the same output, namely the original micrograph placed alongside the outlined image, which is then used by this macro. Clicking on **intervein trichome density** button brings forth a prompt to select an intervein region manually (Figure 3). Doing this by clicking on an intervein region outlines it in yellow, and when “OK” is clicked in the prompt dialog box, the macro uses information from the ROI log to calculate intervein region-specific (1) area in pixels, (2) trichome count, and (3) trichome density (per pixel).
(x) **heat map: intervein regions**
  This button relies on the ROI color coder plug-in (imagejdocu.tudor.lu/doku.php?id = macro:roi_color_coder) to assign heat map colors based on intervein region size determined by **segmentation** analysis (Figure 4).
(xi) **heat map: trichome density**
  This button relies on the ROI color coder plug-in to assign heat map colors based on trichome density determined by Find Maxima > Segmented Particles, which finds each trichome, and draw lines between adjacent trichomes and segments the image by a watershed algorithm; based on the small area subtended, a heat map color is assigned.
(xii) **heat map adjust**
  This button allows users to select either intervien area heat maps or trichome density heat maps and adjust the range of pixels that assign heat map colors and the color scheme as well.

### Availability and requirements

Files that are available at SourceForge (https://sourceforge.net/projects/fijiwings/) include:

(a) A zip file version packaging the entire FijiWings for MacOS complete with (1) the plug-ins ROI color coder and Action Bar and (2) the data.arff file to accomplish the segmentation analysis.
(b) Example photomicrographs of (1) engrailedGAL4 > UAS-Trbl, (2) engrailedGAL4 > UAS-PtenRNAi, and (3) engrailedGAL4 > UAS-Y.
  Instructions for set up are given at this site.
  *Project name* - FijiWings
  *Project home page* - http://sourceforge.net/projects/fijiwings
  *Operating system(s)* - MacOS
  *Programming language* - Java/ImageJ script
  *License* - GNU GPL
  *Tutorial* - http://www.youtube.com/user/Fijiwingsadmin/videos

## Results

Although area measurements of wing size in photomicrographs can be accomplished using simple lasso tools, our efforts to manually count trichomes in defined areas were not only slow and tedious but error prone, for instance, missing rows or double counting rows of trichomes, so we sought an automated tool to measure wing size and the number of trichomes (FijiWings as described in Materials and Methods). Here we describe a series of tests of these tools.

We first examined the ability of the **75px trichome density** macro to detect and count trichomes. As shown in Figure 1, trichomes failed to be counted properly either because they appear to share the same trichome base (lower arrows in each inset) or are too small to be detected (upper arrow in each inset). To examine the frequency with which this occurs, we selected five different 75px regions in three different micrographs and then manually marked trichomes that failed to be counted (Supporting Information, Figure S1). From this we find the **75px trichome density** macro is adept at counting properly from 93.3–98.2% of trichomes visible (data not shown). Images taken in focus at greatest contrast performed better in detecting trichomes than low contrast images. Compared with the frequent errors that we experienced in manually counting 70−80 trichomes in a defined area, we find that the level of accuracy in this automated method was quite tolerable. Precision is 100% so that a macro written to repeatedly count the same area resulted in the same trichome count (data not shown).

To test further the **75px trichome density** function, we used the engrailed GAL4 driver to misexpress the Trbl gene, which has been shown previously to reduce significantly trichome density in the posterior wing compartment (Mata *et al.* 2000). We examined a representative engrailedGAL4 > Trbl wing and measured trichome density in three independent 75px squares taken in the posterior compartment and three in the anterior compartments. The average of the posterior/anterior trichome density ratios for Trbl misexpressing wings was 0.51 compared with 1.10 for WT wings. Thus, the **75px trichome density** function can effectively detect the reduced cell density in wings misexpressing Trbl.

Given the effectiveness of the Process > Find Maxima function to identify individual trichomes in user-defined squares at both high (Figure 1A) and low (Figure 1B) magnification, we sought to determine both trichome number and compartment area in a representative engrailedGAL4 > UAS-Trbl wing. As shown in Figure 4A, the **polygon trichome density** function detected a significant reduction in the posterior/anterior ratio of trichome density in a engrailedGAL4 > UAS-Trbl wing compared with a WT wing (an outcome similar to that seen for the **75px and 150px trichome density** functions) and detected also an overall reduction in posterior/anterior compartment size of a engrailedGAL4 > UAS-Trbl wing compared to WT wing (Figure 4A).

The **polygon trichome density** tool shown in Figure 2 is useful when the compartment boundary is straightforward to detect, however in wings in which this boundary is not readily identifiable, we sought to use the wing veins as landmarks and automate the measurement of trichome density in intervein regions. In representative wings dissected from flies misexpressing Trbl, the Yellow gene (which serves as a WT control), or Pten RNAi (Gao *et al.* 2000), which has been shown previously to increase tissue size and cell number (Tang *et al.* 2011), we used the **segmentation (8 GB)** function to automatically identify and outline veins and wing edges. We then manually selected the posterior-most intervein region (properly called the third posterior cell) and used the macro **intervein trichome density** (Figure 3) to calculate area and trichome density. As shown in Figure 3B and summarized in Figure 4B , segmentation analysis followed by trichome density determination confirms that Trbl reduces both trichome density and tissue size while Pten RNAi increases both relative to WT in this region.

**Figure 5:**
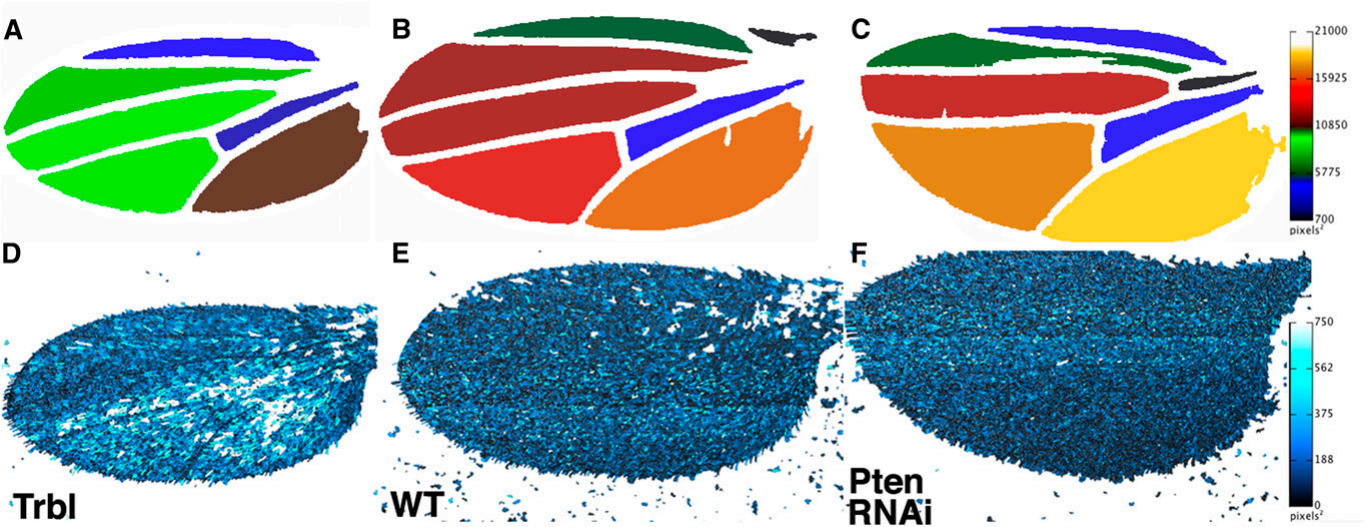
Heat maps of intervein areas and trichome density detect effects of misexpressing Trbl and Pten RNAi. Heat map macros (area, A−C) and (trichome density, D−F) applied to Trbl (A, D), WT (B, E), and Pten RNAi wings (C, F), respectively. Note the decrease in size for the second posterior cell of the posterior compartment expressing Trbl (A) as detect by a red to green change and similarly an orange to brown change to demonstrate the reduction in size for the third posterior cell. Note the complementary increase in size of the posterior compartment expressing Pten RNAi (C) as detect by a red to orange change for the second posterior cell and orange to yellow for the third posterior cell. The heat map trichomes outlines the areas between trichomes and assigns these colors based on size (inset) so that the posterior compartment of wings misexpressing Trbl (D) wings is noticeably lighter than WT (E) and Pten RNAi (F) is noticeably darker, consistent an increase in trichome density from D−F.

The **heatmap interveins** function was used to compare segmentation analysis of wings misexpressing Trbl (Figure 5A), the Yellow gene (WT; Figure 5B), and Pten RNAi (Figure 5C). Compared with WT, the relative reduction in engrailedGAL4 > Trbl posterior wing size area is detectable as a red to green and orange to brown color change for the second and third posterior intervein regions, respectively (*cf*. Figure 5, A and B). However, for engrailedGAL4 > Pten RNAi, the size of posterior wing areas is larger than WT (Figure 5C), which is depicted as orange and yellow second and third posterior intervein regions, respectively, compared to red and orange for the same intervein regions in WT (*cf*. Figure 5, B and C). This confirms in a visual manner the significant increase in tissue size that occurs when Pten levels are reduced. When combined with the increased trichome density noted previously for Pten RNAi (Figure 4B), these results support previous observations that reducing Pten activity increases both cell proliferation and tissue size.

Finally, we compared the density of trichomes in wings expressing Trbl, Yellow, and Pten RNAi by using the **heatmap trichomes** function, which assign a heat map color to the small area subtended by adjacent trichomes. As can be seen in Figure 5, D−F, the **heatmap trichomes** function reveals that the overall density of trichomes throughout the posterior compartment of Trbl-expressing wings is notably reduced, resulting in ‘hotter’ white and light blue colors (Figure 5D) compared with the dark blue associated with WT wings (Figure 5E). In contrast, a darker blue color is associated with Pten RNAi misexpression (Figure 5F). These kinds of heat map data can offer an overall impression of the trichome density across the entire wing and **heat map adjust** allows users to change color schemes and tailor the range of pixels for best presentation of results.

## Discussion

Here we present FijiWings as a tool to precisely measure cell number (represented as trichome count) and tissue size (in pixels) in the Drosophila wing using several integrated functions. The ability of Fiji/ImageJ to detect individual trichomes requires high-resolution, sharply focused photomicrographs of wings that are prepared to be as flat and clear of dust and contaminants as possible. The Process > Find Maxima function detects the slightly darker base of individual trichomes and only fails to count trichomes in two classes: (1) those from the dorsal and ventral side of the wing that coincide and appear to share the same base and (2) those too small to be detected. While reducing the noise setting (possible using the **noise adjust** macro) can detect the latter class (data not shown), dirt and dust are often counted inappropriately. In our hands, FijiWings misses only 4–7% of trichomes that we could detect after a supplemental manual inspection (Figure S1). Because human counting errors include miscounting and double counting both individual trichomes and whole rows of trichomes, we consider this a good error rate for high-throughput approaches.

We tested the ability of FijiWings to measure wing properties by misexpressing transgenes known to influence trichome density and tissue area in the wing. FijiWings effectively detected the ability of Pten RNAi misexpression to increase tissue size and cell number, the latter seen as a slight increase in trichome density (Figure 4B). These outcomes are consistent with previous work showing that Pten is required to reduce both wing cell proliferation and wing cell size by antagonizing insulin signaling (Gao *et al.* 2000). Trbl misexpression led to a detectable decrease in trichome density (Figure 4), which has been reported (Mata *et al.* 2000), however the reduction in overall wing size following Trbl misexpression was not previously noted. The ability of Trbl to reduce the cell cycle likely leads to decreased trichome density, and the effect we see on overall tissue size is consistent with the observation that as the cell cycle slows, so does the rate of cell growth (Neufeld *et al.* 1998). The effect of Pten and Trbl is manifest as well in heat maps of trichome density and intervein areas, each of which gives an overall impression consistent with more precise measurements (*cf*. Figures 4 and 5). In summary, the ability of FijiWings to measure trichome density and tissue size confirms the known effects of Pten on cell proliferation and reveals a new function for Trbl in tissue growth, demonstrating the potential of this tool to identify gene interactions that might only subtly influence cell growth and proliferation phenotypes.

Plug-ins for Fiji/ImageJ may have wider uses to mine high-resolution wing micrographs for accurate measurements of changes in tissue growth in response to environmental and nutritional changes, and to compare the evolution of wing patterns. Subtle phenotypic differences can be detected, for example between WT Trbl misexpression and misexpression of point mutations in the Trbl gene (L.L. Dobens, unpublished data). Although here we only measure trichome density, we have had success outlining the shapes of individual trichomes using the “Analyze Particles” function of Fiji/ImageJ (data not shown), which offers a potential means to catalog the effect of genes that influence trichome shape and arrangement on the surface of the wing, which will be of interest to workers studying cell polarity. The application of the trainable segmentation functions of FijiWings to wing morphometry could be adapted to (1) establish patterns of speciation (Favret 2009; Francuski *et al.* 2009; Muñoz-Muñoz *et al.* 2011; Vicente *et al.* 2011; Oyerinde *et al.* 2012)’ (2) test ideas of plasticity in tissue growth (Hidalgo 1998); and (3) measure variation between populations, sexes (Klingenberg and Gidaszewski 2010; Carreira *et al.* 2011), individuals, and even compartments in the same individual (Klingenberg and Zaklan 2000; Martin and Morata 2006). Because any two-dimensional feature can be analyzed, these tools can be adapted to measure leaf structure (Maloof *et al.* 2013) and nonbiological structures such drainage topography (Ehsani and Quiel 2008).

## Supplementary Material

Supporting Information
