# Supplementary material for: FijiWings: An Open Source Toolkit for Semiautomated Morphometric Analysis of Insect Wings
Source: G3 (Bethesda). 2013 Aug 1;3(8):1443–9. doi: 10.1534/g3.113.006676 (PMC3737183; doi:10.1534/g3.113.006676)
Supplement: Supporting Information [file supp_3_8_1443__index.html]

FijiWings: An Open Source Toolkit for Semiautomated Morphometric Analysis of Insect Wings — Supporting Information 

# FijiWings: An Open Source Toolkit for Semiautomated Morphometric Analysis of Insect Wings

## Supporting Information for Dobens and Dobens, 2013

**Files in this Data Supplement:**

- Figure S1 - Fraction of trichomes detected by FijiWings (PDF, 709 KB)
