# Supplementary material for: FijiWings: An Open Source Toolkit for Semiautomated Morphometric Analysis of Insect Wings
Source: G3 (Bethesda). 2013 Aug 1;3(8):1443–9. doi: 10.1534/g3.113.006676 (PMC3737183; doi:10.1534/g3.113.006676)
Supplement: Supporting Information [file supp_g3.113.006676_006676SI.pdf]

G3: Genes | Genomes | Genetics

FijiWings: an open source toolkit for semi-automated morphometric analysis of insect wings

Alexander C. Dobens and Leonard L. Dobens*

Division of Molecular Biology and Biochemistry
School of Biological Sciences
University of Missouri-Kansas City
Kansas City, MO 64110

*Corresponding author:

Dr. Leonard L. Dobens, Jr.
Division of Molecular Biology and Biochemistry
School of Biological Sciences
University of Missouri-Kansas City
Kansas City, MO 64110
Tel: 816-523-3778
Fax: 816-523-5995
Email: dobensl@umkc.edu

DOI: 10.1534/g3.113.006676

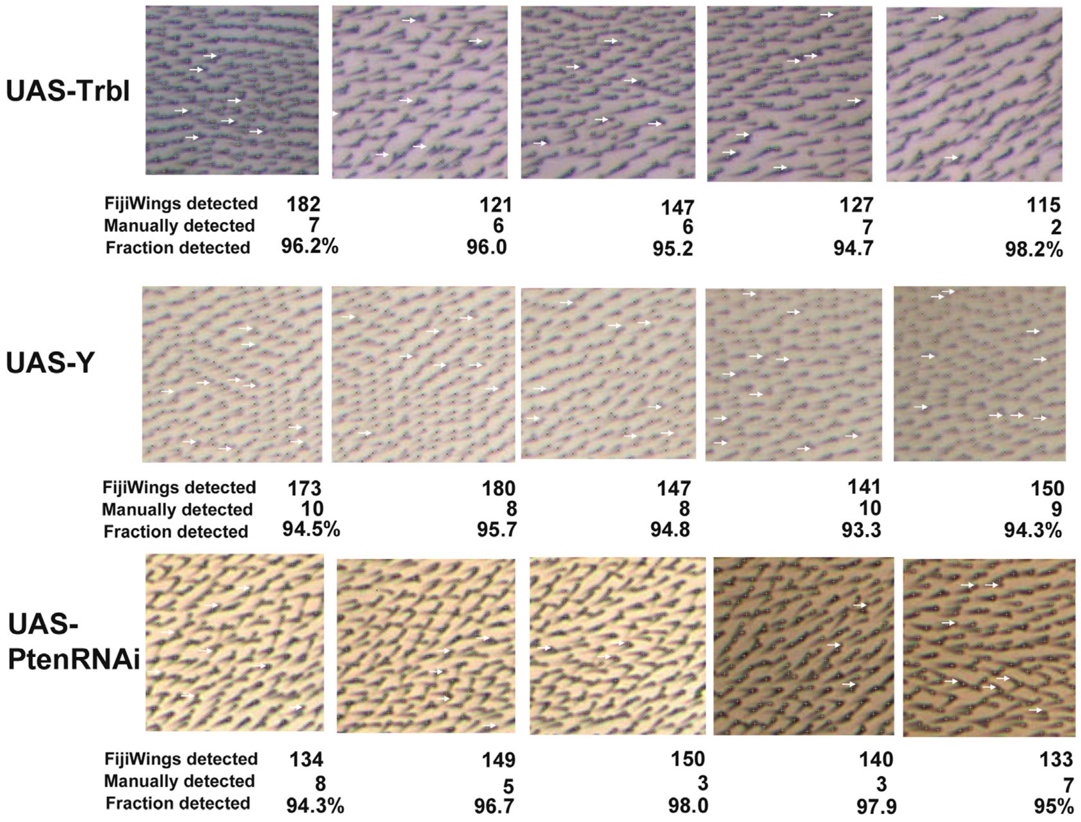

Figure S1 Fraction of trichomes detected by FijiWings. 75px trichome counting tool was used to select five intervein areas (columns) from three different micrographs (rows: YAS-Trbl, UAS-Yello and UAS-PtenRNAi) and trichome counds were recorded (FijiWings detected). Manual inspection of these identified trichomes not detected by automated counting and these were marked (white arrows) and recorded (Manually detected). Fraction detected (as %) = 100 x FijiWings detected/ (FijiWings detected + Manually detected).
